# Surgical management of lateral neck abscesses in children: a retrospective analysis of 100 cases

**DOI:** 10.1007/s00431-022-04676-5

**Published:** 2022-11-15

**Authors:** Nyat-Eyob Tecle, Stephan Hackenberg, Matthias Scheich, Agmal Scherzad, Rudolf Hagen, Thomas Gehrke

**Affiliations:** 1 Department of Otorhinolaryngology, Plastic, Aesthetic and Reconstructive Head and Neck Surgery, University Hospital of Würzburg, Würzburg, Germany; 2 Department of Otorhinolaryngology and Head and Neck Surgery, University Hospital RWTH Aachen, Aachen, Germany

**Keywords:** Lateral neck abscesses, Children, Focus of infection, Surgery

## Abstract

Cervical abscesses are relatively common infections in pediatric patients. There is an ongoing debate about the necessity and time point of surgical drainage. The identification of a focus of infection might play an important role in facilitating a therapeutic decision. In a retrospective study, 100 pediatric patients aged 1–18 years who underwent incision and drainage of a lateral cervical abscess at our institution were analyzed. Patients were divided into two groups based on whether a focus of infection could be identified or not. Data collection included patient characteristics, microbiological results, antibiotic regimen, and clinical course. A focus of infection was found in 29% (29/100) of the patients, most frequently in the tonsils. A causative microorganism was found in 75% (75/100) of all patients, with *Staphylococcus aureus* and *Streptococcus pyogenes* being the most common pathogens. All patients received an empiric antibiotic therapy in addition to surgery. Antibiotic medication was changed in 31% in both groups (9/29 with a focus of infection and 22/71 without a focus of infection) during therapy. Children without an identified focus of infection generally were younger and had more comorbidities reducing immune response while also showing differences in the pathogens involved. There were no complications associated to surgery or antibiotic therapy in any of the patients involved.

*Conclusion*: Children with an identified focus of infection show several differences compared to those with isolated lateral abscesses, especially regarding the microorganisms involved. But the focus of infection seems not to have an impact on patient’s outcome.

**Table:** 

**What is Known:** *• Neck abscesses are a relatively common disease in the pediatric population and may cause serious complications.* *• Therapy in general consists of intravenous antibiotics with or without surgery.*	
**What is New:** *• The focus identification has no impact on patient’s outcome.* *• Children with an identified focus of infection show several differences compared to those with isolated lateral abscesses, especially regarding their medical history, age, and the microorganisms involved.*

**What is Known:**

*• Neck abscesses are a relatively common disease in the pediatric population and may cause serious complications.*

*• Therapy in general consists of intravenous antibiotics with or without surgery.*

**What is New:**

*• The focus identification has no impact on patient’s outcome.*

*• Children with an identified focus of infection show several differences compared to those with isolated lateral abscesses, especially regarding their medical history, age, and the microorganisms involved.*

## Introduction

Cervical abscesses are a relatively common disease in the pediatric population. They often present with neck swelling, erythema, reduced oral intake, and fever [1], but the initial diagnosis may be delayed because of the poor verbal communication and malcompliance regarding a comprehensive head and neck physical examination [2]. For evaluating these patients, in most cases, ultrasound and computed tomography or magnetic resonance imaging are used to confirm abscess formation [3–5]. While deep neck infections in adults often present with an odontogenic focus of infection, the origins for pediatric neck abscesses are usually upper respiratory tract infections such as rhinosinusitis, tonsillitis, pharyngitis, and suppurative cervical adenitis [6]. As a result, aerobic bacteria like *Staphylococcus aureus* and *Streptococcus pyogenes* are the most common isolated pathogens, while anaerobic bacteria are found less frequently [6, 7]. Although the clinical course of cervical abscesses in children is relatively moderate compared to adults, deep neck abscesses may cause serious complications including airway obstruction, jugular vein thrombosis, and mediastinal dissemination and consequently possess a potential to lead to significant disease-related morbidity and mortality [8, 9]. Moreover, complications are more frequent when there is a pre-existing immunosuppression or a delay in diagnosis and treatment [10]. The presence of toothache and neck pain on admission was also identified as possible predictors of complications [11]. The optimal management of cervical abscesses is still subject of ongoing debates but generally consists of intravenous antibiotics [12, 13] and surgical drainage [14, 15]. Surgical incision and drainage have been the mainstay of treatment [16–18], as they do not only provide an immediate relief of the suppurative focus, but also give important microbacterial and histopathological information to adapt antibiotic therapy [16, 19–21]. However, several recent studies advocate a more conservative therapeutic approach including a trial of parenteral antibiotic therapy coupled with close clinical observation [22, 23]. Previously delineated risk factors for surgical drainage, which have been mentioned as the rationale behind conservative approaches, include abscess size, age, positive inflammatory markers, intensive care status, and prior emergency department visits [6, 9, 24]. Recent reports, however, describe a remarkable rise in the incidence and complications of pediatric cervical abscesses [25, 26]. Some authors propose a relation of this trend with the emergence and increasing incidence of more invasive pathogens such as methicillin-resistant *Staphylococcus aureus* (MRSA) and growing resistance to traditionally employed antibiotic regimens [27, 28]. Another theory on the etiology of recalcitrant pediatric neck abscesses is a biofilm formation as a possible mechanism behind ineffective primary antibiotic management [29]. While a correlation between the increasingly conservative management and an increase in complicated clinical courses of pediatric cervical abscesses might be postulated, there is no sufficient evidence in literature, especially regarding children. Hence, more ways to help facilitate the decision between conservative and surgical therapy in these patients would be valuable.

Since most deep neck abscesses have their origin in the head and neck region, a physical examination particularly by an otorhinolaryngologist to find a focal point seems reasonable but is not regularly provided. So far, no study has examined whether the identification of an infectious focus has an impact on the clinical course.

Therefore, the aim of this retrospective analysis is to evaluate the perioperative outcome of children with cervical abscesses receiving surgical drainage depending on whether an infectious focus could be identified or not and thus to provide additional information to base future therapeutic decisions on.

## Methods

After receiving approval of the Würzburg University’s Hospital Institutional Review Board and local ethics committee in October 2019 (#20,191,004 02), a retrospective chart review of all patients aged 18 or younger who underwent incision and drainage of a sonographically confirmed lateral cervical abscess at our institution from January 2009 to December 2019 was performed. The study comprised all consecutive children presenting with a lateral neck abscess at our institution in that period. Patients older than 18 years, with an isolated peritonsillar abscess or exclusive intravenous antibiotic therapy were excluded. Altogether, 100 patients could be identified who met the inclusion criteria, and those were thereafter divided into two groups based on whether a focus of infection could be identified or not. Patient characteristics investigated included age, gender, and medical history like immunosuppressive or syndromal diseases. “Immunosuppressive disease” means a certain disease or condition that is associated with a weakened immune system, and therefore, these patients might be more susceptible to develop infections like a neck abscess. In our study, we considered chronic granulomatosis, leukemia, lymphoma, anemia, thalassemia, and prematurity to be immunosuppressive diseases. Blood values, especially leukocytes and C-reactive protein (CRP) were also evaluated. Besides the clinical examination, ultrasound was performed in all children to verify abscess formation. Furthermore, the ultrasound exam was performed and interpreted by an otorhinolaryngologist and a pediatric radiologist in order to increase the accuracy of the diagnosis. Only a select few patients received an additional MRI in case of uncertain sonographic results. All patients were treated with an initial empiric antibiotic therapy consisting of a penicillin in combination with a ß-lactamase inhibitor (such as ampicillin with sulbactam) or a ß-lactamase-resistant antibiotic (such as cefuroxime or cefotaxime) in combination with clindamycin or metronidazole. Furthermore, all patients underwent incision and drainage of the neck abscess in general anesthesia the same day as admission. During surgery, small drainage tubes were placed and rinsed with antiseptic fluids and sodium chloride twice a day. After removal of the drainage tubes, the neck wound was left to secondary wound closure, which was successful in all but one patient who needed a surgical closure. Purulent material as well as lymph node tissue was also obtained during surgery in order to determine the causative pathogen and to exclude an underlying malignant disease. Microbiologic and pathological results were analyzed in all patients. Besides the antibiotics administered, a change of substance during therapy as well as the overall duration of antibiotic therapy was analyzed. The frequency of surgical interventions, the duration of drainage tubes, the complications of surgery, and the length of hospital stay were also investigated. The results of both groups were compared and examined for significant differences.

Data collected were transferred to standard spreadsheets and statistically analyzed using GraphPad Prism software (version 6.0e, GraphPad Software Inc., San Diego, CA, USA). D’agostino–Pearson omnibus normality test was conducted to verify normality distribution.

Due to the normal distribution, the Students *t* test was applied when comparing two groups with continuous variables. Categorical data were analyzed with Fisher’s exact test or Chi-squared test for more than 2 variables. A value of *p* < 0.05 was considered statistically significant. When testing multiple comparisons, a Bonferroni correction was applied to each group of tests.

## Results

**Fig. 1:**
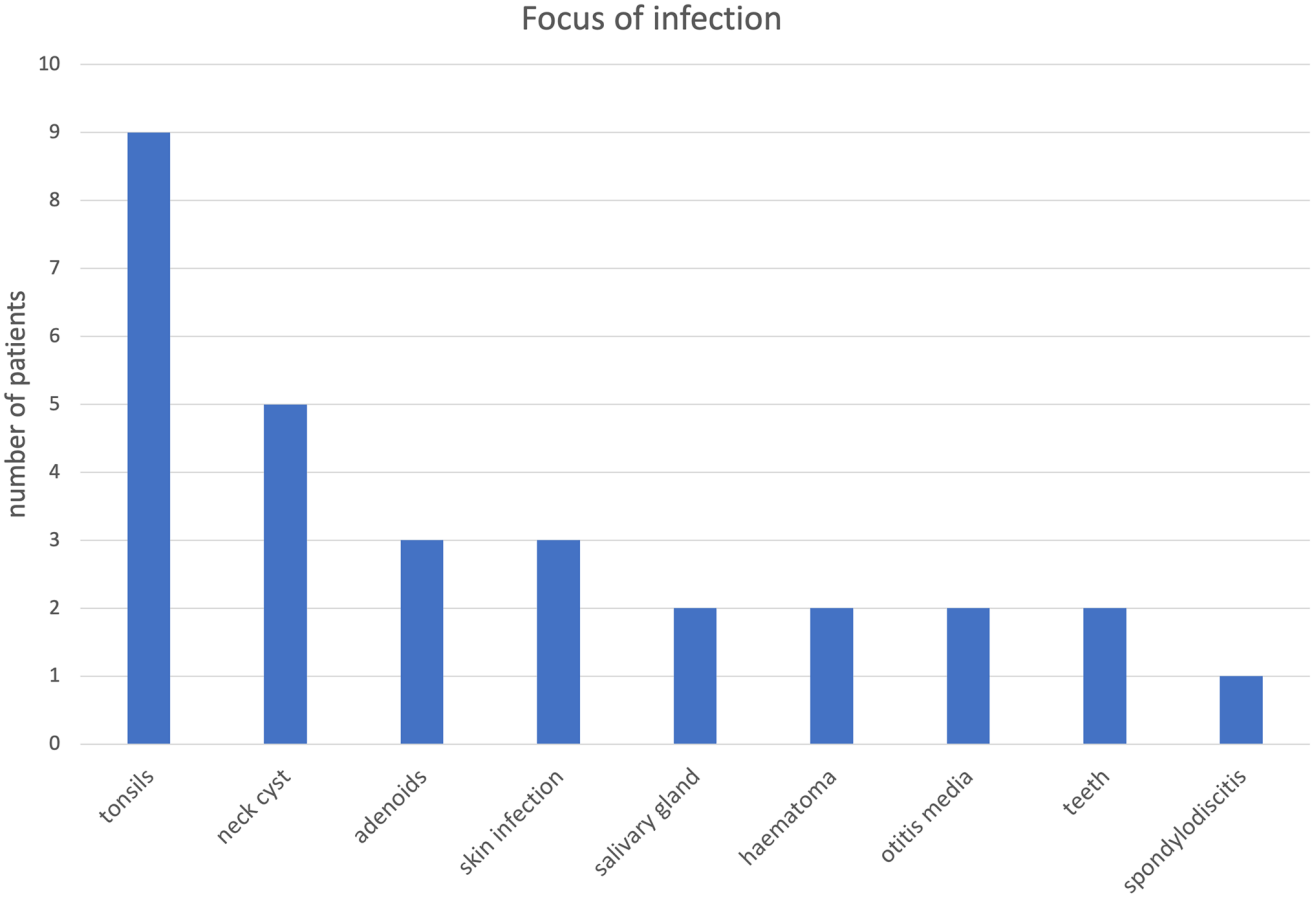
Summary of the identified sources of infection in 29 patients

A total of 100 patients who met the inclusion criteria were analyzed. In 29 patients, a focus of infection was found, most frequently the tonsils (9 patients, 31.0%), neck cysts (5 patients, 17.2%), adenoids, and skin infections (3 patients each, 10.3%). Neck cysts comprise congenital masses in the neck including branchial cleft cysts and thyroglossal duct cysts. Figure 1 provides a summary of the identified sources of infection in 29 patients.

Patient characteristics and initial findings in patients with and without a known focus of infection are shown in Table 1.

**Table 1:** Patient characteristics and initial findings in patients with and without a focus of infection

	Focus of infection (*n*, %)	No focus of infection (*n*, %)	*p* value
Total number of patients	29 (29.0)	71 (71.0)	
Gender			0.077
Male	11 (37.9)	42 (59.2)	
Female	18 (62.1)	29 (40.9)	
Age in years	5.97 (0–18)	3.34 (0–18)	*0.004
Immunosuppressive disease	1 (3.5)	15 (21.1)	*0.035
Initial inflammation values			
Leukocytes in 1000/μl	15.26 (4.8–26.88)	15.27 (3.78–34.2)	0.993
CRP in mg/dl	3.87 (0.008–10.16)	3.44 (0.03–15.6)	0.519

Values are given in numbers (proportion) or mean (range)

*Indicates significance after Bonferroni correction

**Fig. 2:**
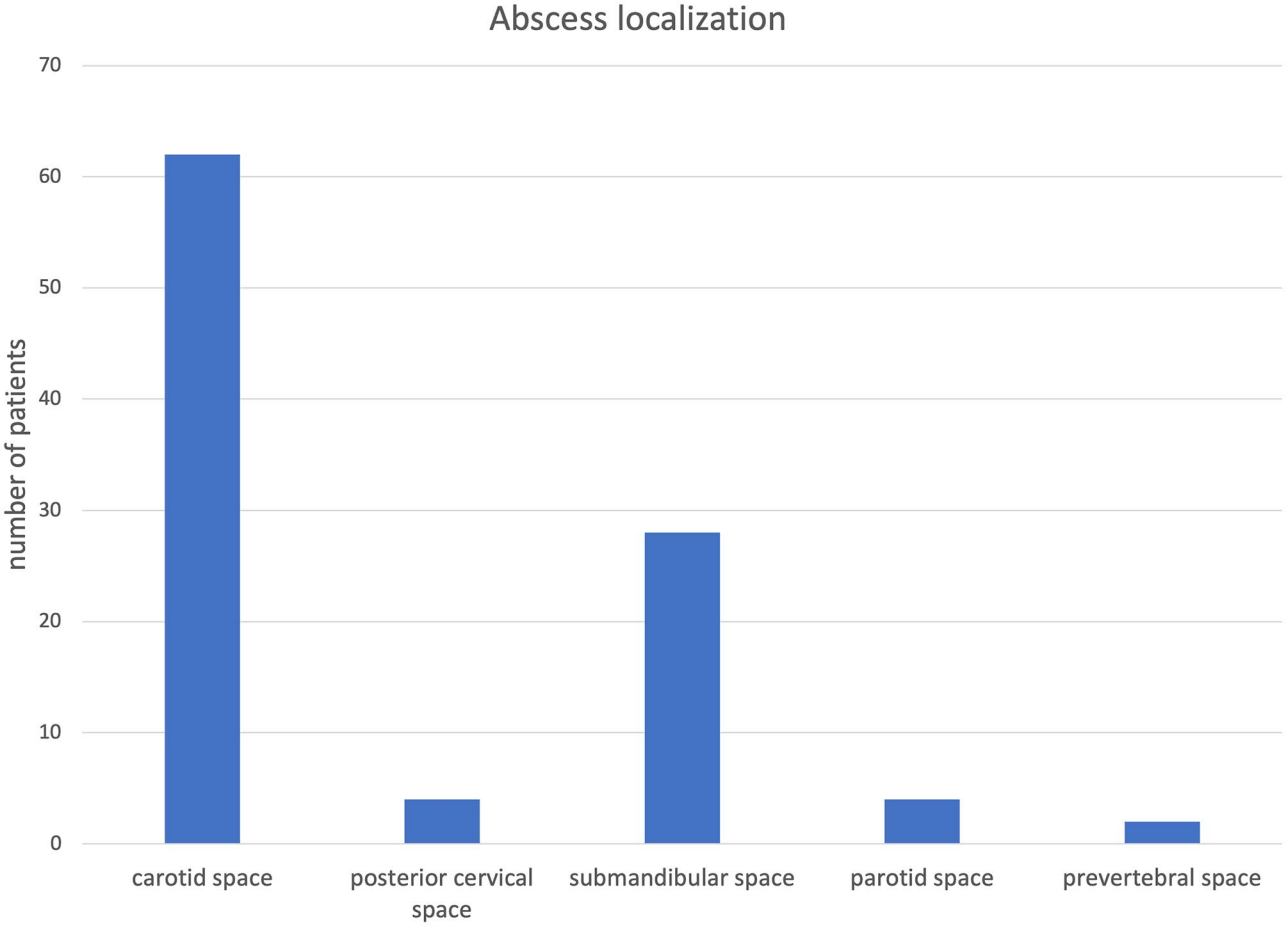
The distribution of the abscess localizations in the examined patients

Male-to-female ratio was 37.9/62.1% vs. 59.2/40.9% (*p* = 0.077). Patients without an identified focus of infection were significantly younger (3.34 vs. 5.97 years, *p* = 0.004) and more often had an underlying immunosuppressive disease (21.1 vs. 3.5%, *p* = 0.035). Initial blood sampling revealed a leukocyte count of 15.260/μl vs. 15.270/μl (*p* = 0.993) and C-reactive protein (CRP) of 3.87 mg/dl vs. 3.44 mg/dl (*p* = 0.519). Figure 2 depicts the distribution of the abscess localizations in the examined patients.

Most abscess formations were found in the carotid space (62 patients, 62%) followed by the submandibular space (28 patients, 28%), while fewer children had involvements of the posterior cervical space (4 patients, 4%), parotid space (4 patients, 4%), and prevertebral space (2 patients, 2%).

The results of bacterial sampling are presented in Table 2.

**Table 2:** Microbacterial results in patients with and without a focus of infection

	Focus of infection (*n*, %)		No focus of infection (*n*, %)	
*ß-Hemolytic streptococci*	9	31.0	13	18.1
*Streptococcus viridans*	1	3.5	8	11.3
*Staphylococcus aureus*	4	13.8	25	35.2
*Coagulase-negative Staphylococcus*	1	3.5	0	0
*Mycobacteria*	0	0	5	7.0
Multidrug-resistant organism (MDRO)	1	3.5	1	1.4
Polymicrobial	0	0	4	5.6
Other	7	24.1	7	9.9
No bacterial growth in culture	9	31.0	16	22.5

Values are given in numbers and proportion. Proportions can exceed 100% due to polymicrobial infections

As expected, the predominantly isolated pathogens in both groups were *ß-hemolytic Streptococci*, *Streptococcus viridans*, and *Staphylococcus aureus*, with a trend for more infections caused by *Streptococcus viridans* in patients without an identified focus and by *Staphylococcus aureus* in patients with a known focus of infection. Contrary to adult populations, polymicrobial infections were generally rare and could only be identified in 3 patients without a known source of infection. Also, the rate of multidrug-resistant organisms was very low, with only one case in each group (3.5% vs. 1.4%).

Mycobacteria were solely found in patients without a focus of infection (5 patients, 7.0%) as well, with 4 cases of mycobacterium avium and one case of tuberculosis. No bacterial growth from the samples occurred in 31.0% of patients with a known focus of infection and in 22.5% of patients without.

Furthermore, in our study, the recurrence rate of abscesses was 10%, as 10 out of 100 patients required a second surgery. Infectious focus could be determined in half of the patients in this group, compared to only 26.7% (24/90) in the group without recurrent abscess. The majority of patients with a recurrence had an infection with mycobacterium other than tuberculosis (MOTT) or an abscess based upon neck cysts.

Histopathologic samples were also taken regularly and led to the identification of a B cell lymphoma in one child.

The diagnostic measures, therapy, and clinical course of the patients involved in the analysis are given in Table 3.

**Table 3:** Diagnostic, therapy, and clinical course of patients with and without a focus of infection

	Focus of infection	No focus of infection	*p* value
Diagnostic imaging			
Ultrasound	29 (100%)	71 (100%)	
MRI	3 (10.3%)	3 (4.2%)	
CT	0	1 (1,4%)	
Hospitalization in days	9.48 (4–37)	8.11 (3–19)	0.128
Antibiotic therapy in days	9.52 (4–37)	8.04 (3–19)	0.104
Change of antibiotic therapy	9 (31.0%)	22 (31.0%)	1.000
Wound lavage in days	3.79 (2–6)	3.34 (2–6)	0.068

Values are given in numbers (proportion) or mean (range)

There were no significant differences between the patients with and without a focus of infection regarding duration of the antibiotic therapy (9.52 vs. 8.04 days, *p* = 0.104), hospitalization (9.48 vs. 8.11 days, *p* = 0.128), and the duration of wound lavage needed (3.79 vs. 3.34 days, *p* = 0.068). Antibiotic regimen was changed in almost one third of patients both with and without a known source of infection over the course of therapy, mostly deescalating after receiving the antibiogram of the microorganism identified. Sixty-seven percent of all patients received a prior antibiotic outpatient treatment, while in 16% of the cases, the intravenous antibiotic therapy was started simultaneously with the surgery. In the remaining 17% of the patients, no data on prior therapy was available.

For imaging of the neck abscesses, ultrasound was the diagnostic standard, and only in rare occasions, magnetic resonance imaging was used additionally. In only one case, a CT scan was performed.

Surgery consisted in incision and drainage of the abscess formation in general anesthesia and the application of silicone tubes for postoperative wound lavage. The drainage tubes could be removed at latest after 3 days of lavage. In case of an identified focus of infection, appropriate measures were taken in order to achieve infectious source control. In total, 5 of the 9 children with a focus in the tonsils underwent a tonsillectomy due to a severe peritonsillar phlegmonous infection, while the other 4 only had an acute tonsillitis covered by the antibiotic regime. Neck cysts as origin of infection were not resected completely during the acute infection but were surgically removed a few weeks after the infection to reduce the risk of recurrence. The 3 patients with infected adenoids received an adenoidectomy, and both children with an acute purulent middle ear infection had a paracentesis to relieve the infectious focus. Patients with an odontogenic focus received a consultation and, if needed, therapy by a dentist. Children with salivary gland infection, skin infections, or infected hematoma as source of infection did not need a specific treatment except the drainage of the neck abscess.

There were no complications associated to surgery or antibiotic therapy, except for one patient without a focus of infection who needed a surgical closure because of a wound healing disorder.

## Discussion

Despite the advances in diagnostic modalities and effective antimicrobial treatment, pediatric neck abscesses remain a serious medical condition with potentially life-threating complications [4, 30] and recently increasing incidence [31, 32]. Cervical abscesses are primarily of oral, nasal, otitic, and odontogenic origin but also caused by infections of salivary glands and congenital cysts [33]. Nevertheless, in children, it is not uncommon to find no primary source of infection [1]. In the present study, a focus of infection was identified in only 29 of 100 patients.

The involved fascial spaces were connected to the origin of infection, as the infections spread to deep neck spaces by direct continuity or by lymphatic drainage to lymph nodes in these areas. For example, dental infections tend to spread through the submandibular space, tonsillar infections commonly follow the parapharyngeal and carotid spaces, and salivary abscesses drain into the parotid, submandibular, and carotid spaces [6]. Therefore, abscess formation in our study was mainly localized in carotid, submandibular, and parotid spaces.

Previous authors [34, 35] already noted that abscesses often yield microflora that are indigenous to this region. Accordingly, the predominantly isolated pathogens in most of the literature as well as in the present study are *ß-hemolytic Streptococci*, *Staphylococcus aureus*, and *Streptococcus viridans* [1, 8, 33], with a trend for more infections caused by *Streptococcus viridans* in patients without an identified focus and by *Staphylococcus aureus* in patients with a known focus of infection in our collective. In contrast to several studies [33, 34, 36], polymicrobial infections with only 3 cases (4,2%) in patients without a known source of infection were very rare. Also, infections by multidrug-resistant organism were noted only in one patient each group (1.4% and 3.5% respectively), which is lower than the proportions reported in literature with 12–50% [10, 28] and might be explained by regional or demographic differences. However, the relatively high proportion of patients with no bacterial growth in both groups (31,0% vs. 22,5%) is in accordance with the majority of studies regarding this topic [33, 37] and might be related to prior antibiotic outpatient treatment, as 67% of the patients in the present study received oral or intravenous antibiotics prior to admission to our institution.

The current trend in the management of pediatric deep neck abscesses involves a trial of intravenous antibiotics prior to consideration of surgical therapy [6, 9, 22, 23], but there are several studies [1, 18, 38] advocating incision and drainage of the abscess coupled with intravenous antibiotics as the effective treatment in order to prevent severe complications.

At our institution, all patients with sonographically proven cervical abscess received surgery on the day of admission along with intravenous antibiotics. In case of an identified focus of infection, additional measures were taken in order to achieve infectious source control.

In the current study, there were no complications such as mediastinitis, internal jugular vein thrombosis, or airway obstruction, although according to the literature, the complication rate ranges between 6 [9] and 10% [1]. Risk factors associated with increased likelihood of developing complications are predominantly younger age, retropharyngeal abscess location, and pre-existing immunosuppression [6, 8, 10].

In the present study, patients without an identified focus of infection were significantly younger and more often had an underlying immunosuppressive disease, yet no complications occurred.

A possible explanation might be prompt surgery. For example, de la Cuesta et al. [17] conducted a retrospective study on the treatment of deep neck abscesses in children over a 15–year period and reported that an early cervicotomy decreased the number of serious complications, whereas Cramer et al. [39] could not find a correlation between timing of drainage and abscess-specific morbidity and mortality in children by performing a multicenter, prospective, risk-adjusted cohort study of adult and pediatric patients with deep neck abscesses who received incision and drainage. In addition, a study published by Velhonoja et al. [40] could show that early surgical intervention is associated with a decreased hospitalization among severe pediatric deep neck infections when comparing the early surgical intervention group to the late surgery group.

The fact that multidrug-resistant organisms were barely present in our study might be another reason for the absence of complications in the present study. Wright et al. [27], for instance, reported that 75% of children with positive MRSA cultures from retropharyngeal abscesses developed mediastinitis. The authors concluded that MRSA is a more invasive pathogen with greater potential for complications compared to other bacterial isolates.

We acknowledge that our present study is limited by its retrospective nature and missing control group as all included patients received surgery. On advantage of this study is the standardized diagnostic and patient management as well as the completeness of data.

In conclusion, the present study showed in a large retrospective analysis that patients without an identified focus of infection show distinct differences regarding patients characteristics like age and microbiological results compared to those with a known focus of infection, although there was no difference in patient’s outcome. Further research is warranted in order to identify those who are at risk to develop complications in time and to work out standardized protocols for managing pediatric patients with deep neck abscesses.
